# Comparative Evaluation of Different Test Combinations for Diagnosis of *Mycobacterium avium* Subspecies *paratuberculosis* Infecting Dairy Herds in India

**DOI:** 10.1155/2015/983978

**Published:** 2015-04-05

**Authors:** Rajni Garg, Prasanna Kumar Patil, Shoor Vir Singh, Shukriti Sharma, Ravi Kumar Gandham, Ajay Vir Singh, Gurusimiran Filia, Pravin Kumar Singh, Sujata Jayaraman, Saurabh Gupta, Kundan Kumar Chaubey, Ruchi Tiwari, Mani Saminathan, Kuldeep Dhama, Jagdip Singh Sohal

**Affiliations:** ^1^Animal Disease Research Centre, Guru Angad Dev Veterinary and Animal Sciences University (GADVASU), Ludhiana, Punjab 141004, India; ^2^Aquatic Animal Health Division, Central Institute of Brackishwater Aquaculture (CIBA), 75 Santhome High Road, R.A. Puram, Chennai 600028, India; ^3^Microbiology Laboratory, Animal Health Division, Central Institute for Research on Goats, Makhdoom, P.O. Farah, Mathura, Uttar Pradesh 281122, India; ^4^Department of Veterinary Medicine, Guru Angad Dev Veterinary and Animal Sciences University (GADVASU), Ludhiana, Punjab 141004, India; ^5^Division of Animal Biotechnology, Indian Veterinary Research Institute, Izatnagar, Bareilly, Uttar Pradesh 243122, India; ^6^National JALMA Institute for Leprosy and Other Mycobacterial Diseases (NJIL & OMD), Tajganj, Agra, Uttar Pradesh 282001, India; ^7^Department of Microbiology, King George's Medical University, Lucknow, Uttar Pradesh 226003, India; ^8^Amity Institute of Microbial Technology, Amity University Rajasthan, Kant Kalwar, NH-11C Delhi-Jaipur Highway, Jaipur 303 002, India; ^9^Department of Veterinary Microbiology and Immunology, Uttar Pradesh Pandit Deen Dayal Upadhayay Pashu Chikitsa Vigyan Vishwa Vidyalaya Evam Go-Anusandhan Sansthan (DUVASU), Mathura, Uttar Pradesh 281001, India; ^10^Division of Pathology, Indian Veterinary Research Institute, Izatnagar, Bareilly, Uttar Pradesh 243122, India

## Abstract

A total of 355 cows were sampled (serum, *n* = 315; faeces, *n* = 355; milk, *n* = 209) from dairy farms located in the Punjab state of India. Faeces and serum/milk samples were screened by acid fast staining and “indigenous ELISA,” respectively. IS*900* PCR was used to screen faeces and milk samples. Bio-load of MAP in dairy cows was 36.9, 15.6, 16.3, and 14.4%, using microscopy, serum ELISA, milk ELISA and milk PCR, respectively. Estimated kappa values between different test combinations: serum and milk ELISA, faecal microscopy and faecal PCR, milk ELISA and milk PCR, faecal PCR and serum ELISA were 0.325, 0.241, 0.682, and 0.677, respectively. Estimation of the relative sensitivity and specificity of different tests in the present study indicated that “serum ELISA” and “milk ELISA” were good screening tests, add “milk PCR” was “confirmatory test” for MAP infection. Combination of milk ELISA with milk PCR may be adopted as a model strategy for screening and diagnosis of JD in lactating/dairy cattle herds in Indian conditions.

## 1. Introduction

Johne's disease (JD) is a chronic progressive incurable granulomatous enteritis of ruminants caused by* Mycobacterium avium* subspecies* paratuberculosis* (MAP). Indian studies showed consistently high bio-load of the disease in large ruminants [1]. Production losses occur mainly through low per animal productivity (loss in milk yield, decline in body weights, diarrhea, reduced fertility, increase in mastitis cases, and emaciation) [2, 3]. Recovery of MAP from human breast milk [4] and gut [5] increased the concern for the control of disease in animals. Diagnosis is difficult due to prolonged incubation, intermittent shedding of MAP, variability in host immune response, fastidious nature, and high cost of imported kits [6]. Early and accurate diagnosis is the prerequisite for the control of infection thereby improving the sustainability and productivity of Indian dairy herds [7]. Reports on the bio-load of MAP using milk as the test sample are scanty [8]. Study aimed to evaluate sensitivity and specificity of different diagnostic tests to estimate bio-load of MAP in dairy herds in Indian husbandry conditions.

## 2. Materials and Methods

### 2.1. Samples and Tests

Thirty dairy farms located in different parts of Punjab state (India) were randomly selected and from each farm a minimum of 10 cows were sampled for blood, faeces, and milk. Between 2005 and 2006, 355 cows were sampled and 315 blood, 355 faeces, and 209 milk samples were collected. Faeces and serum/milk samples were screened by acid fast staining and “Indigenous ELISA” tests, respectively. IS*900* PCR was employed for screening of faecal and milk samples. Combinations of serum ELISA and milk ELISA, faecal microscopy and faecal PCR, milk ELISA and milk PCR, and faecal PCR and serum ELISA were applied on 204, 68, 207, and 62 dairy cows, respectively, to evaluate best combination for the diagnosis of Johne's disease in dairy cattle herds. Whey was curdled with 3.0% citric acid and centrifuged at 5000 rpm for 30 minutes to collect clear whey, which was stored at −20°C till further use for the presence of MAP antibodies by “Indigenous ELISA Kit” [9].

### 2.2. Indigenous ELISA Kit

ELISA was performed on serum and milk samples as per Singh et al. [8, 10]. Briefly, serum and whey were diluted in dilution buffer (PBST containing 1% BSA) in the ratio of 1 : 50 and 1 : 10, respectively. The 100 *μ*L of diluted serum and whey was poured to each well (precoated with antigen) in duplicates for 2 hrs at 37°C. Three washings were given after 2 hrs of incubation with PBST. The 100 *μ*L of 1 : 8000 rabbit anti-bovine horse radish peroxidase conjugate diluted in PBS was added to each well and incubated at 37°C for an hour. Plates were washed 3 times (5 min each) with PBST. Finally, 100 *μ*L of freshly prepared substrate (OPD) was added to each plate (pH 5.0). Plates were incubated in dark for 20 minutes at room temperature. Positive (infected) and negative (healthy) serum and whey controls were used with each plate. Absorbance was read at 450 nm in ELISA reader (Labsystem, Finland). S/P ratio was calculated as per the method of Collins [11] by equation: S/P ratio = (OD of sample − OD of negative control)/(OD of positive control − OD of negative control). Interpretation: OD values of 0.00–0.09 as negative, 0.10–0.24 as suspected or borderline, 0.25–0.39 as low positive, 0.40–0.99 as positive, and 1.00–10.0 as strongly positive.

### 2.3. DNA Extraction and PCR

Briefly, fat and sediment layers were dissolved in lysis buffer (10 mm Tris-Cl, 10 mM EDTA, 0.5% Tween 20, 0.5% TritonX 100, 1 M guanidium isothiocyanate (GITC); 0.3 M sodium acetate, pH 7.5) and were processed to DNA extraction as per van Embden et al. [12] with some modifications. Purified DNA was amplified by PCR using IS*900 *based sequence (IS*900 *S2/R1) [13]. Briefly, in a volume of 50 *μ*L of reaction mix that consisted of 10 mM dNTPs mix, 20 pM of each primer, 1.5 Units of Taq polymerase, 25 mM of MgCl_2_, 10x Taq buffer, and 5 *μ*L of extracted DNA was added. Amplification conditions were initial denaturation at 94°C for 3 min, 45 cycles of denaturation at 94°C for 45 sec, annealing at 55°C for 45 sec, and extension at 72°C for 45 sec, and the final extension was done at 72°C for 10 min. MAP specific amplified PCR product (450 bp) was seen in 1% agarose ethidium bromide gel electrophoresis and purified using QIA quick gel extraction kit (QIAGEN, Germany).

### 2.4. Nucleotide Sequence Analysis

Purified PCR product was sequenced by cycle sequencing at Department of Bio-chemistry, Delhi University (South Campus), New Delhi. Nucleotide sequence was analysed using UPGMA, Neighbour-Joining, and Minimum evolution programs in the Mega 3.1 [14]. Sequencing was carried out to confirm the identity of amplified products obtained from different cows using IS*900* PCR on milk samples vis a vis fully sequenced MAP K10 (ACCESSION number EF015397).

### 2.5. Statistical Analysis

Agreement between test combinations (serum ELISA–milk ELISA, faecal microscopy–faecal PCR, milk ELISA–milk PCR, and faecal PCR–serum ELISA) were compared by kappa index test [15] (<0.20, poor; 0.21–0.40, fair; 0.41–0.60, moderate; 0.61–0.80, substantial (good); and 0.81–100, almost perfect). Furthermore, relative sensitivity (the ratio of true positive results to the total of true positives and false negatives) and specificity (the ratio of true negatives results to the total of true negatives and false positives) at 95% confidence interval were also calculated.

## 3. Results

Bio-load of MAP infection in dairy herds using microscopy, serum ELISA, milk ELISA and milk PCR was 36.9, 15.6, 16.3, and 14.4 percent, respectively (Table 1). Of 204, 68, 207 and 62 cows screened by serum ELISA-milk ELISA, faecal microscopy–faecal PCR, milk PCR–milk ELISA, and serum ELISA–faecal PCR test combinations, 7.8, 30.9, 11.1, and 40.3 percent cows were positive, respectively (Table 2). Cows negative in two test combinations were highest as compared to number of cows in any other test combination (positive in both test or positive in one test and negative in another or vice versa) (Table 2). Diagnosis was confirmed by amplification of MAP genomic DNA from faecal and milk samples using IS*900 *PCR. Sequencing of the amplified product (450 bp) confirmed exact location of amplification and sequences were exactly identical to IS*900 *sequences of MAP K10 (ACCESSION number EF015397). Value of kappa coefficient was lowest (0.241), between faecal microscopy-faecal PCR indicating “fair agreement”. Whereas highest kappa value (0.682) with “substantial/good agreement” was reported in milk ELISA–milk PCR (Table 3). In test combinations (serum ELISA–faecal PCR, milk ELISA–milk PCR, and faecal microscopy–faecal PCR) relative sensitivity of serum ELISA (0.833), milk PCR (0.793), and faecal microscopy (0.677) was higher than serum ELISA–Milk ELISA test combination (0.470) (Table 3). In the test combinations relative specificity of Milk ELISA–milk PCR was highest (0.938) as compared to serum ELISA–Milk ELISA (0.870), serum ELISA–faecal PCR (0.844), and faecal microscopy–faecal PCR (0.500).

## 4. Discussion

Infection of MAP in animals and human beings lead to inflammation of intestines and mesenteric lymph nodes [16] and MAP is known to persist in harsh microenvironment inside activated macrophages [17] causing chronic granulomatous enteropathy characterized by persistent diarrhoea, emaciation and death in large ruminants. MAP is not limited to ruminants but expansion of hosts from polygastric to monogastric and from monogastric to carnivores and wild ruminants therefore MAP infection in animals exhibit wide host range and complex epidemiology. Isolation of MAP from intestinal biopsies of Crohn's disease patients led to the concern that it may be a potential zoonosis [18]. Control of disease in India is hindered by the presence of large number of cases of disease in domestic livestock species, lack of information on strain diversity and non-availability of “indigenous kits” for early detection. For diagnosis culture (feces, milk, and intestinal tissues), ELISA and PCR have been routinely used. Long incubation (12–16 weeks) and low sensitivity (>50%) limit the use of culture [19]. Utility of serological tests is limited due to low specificity and sensitivity, as immune response may not be detectable either due to anergy or late appearance in pathogenesis. For the control of disease in dairy herds it is essential to know frequency and distribution of MAP infection. Studies on prevalence have been used to identify and quantify management factors that may be associated with the disease.

“Indigenous ELISA” has been standardized in our laboratory since 2004 using likelihood ratio method [11]. On the basis of percent detection of MAP (Table 1), sensitivity of microscopy was highest followed by milk ELISA, serum ELISA, and milk PCR in the present study. Faecal microscopy has been reported to be cost effective, easy to perform, repeatable, and convenient test for screening of domestic ruminants as compared with blood PCR [20]. However, milk culture has been reported to be most sensitive followed by milk ELISA and milk PCR [21]. But “Indigenous milk ELISA” has been reported to be most sensitive, fast and inexpensive test for large scale screening of lactating goats in endemic regions as compared to milk microscopy and milk PCR [22]. In early and subclinical stages of infection in goats as compared to faecal microscopy and serum ELISA, blood PCR was rapid, sensitive and specific [23]. Though sensitivity and specificity of microcopy was low [24], it helped in estimating rate of shedding of MAP in faeces of infected animals. However, negative results do not rule out MAP infection [25]. Success of the test depends upon the number of bacilli present in sample [26]. Due to poor sensitivity, results may not reflect true prevalence of disease as it detects only clinical shedders [27]. Though culture is most sensitive and specific (gold standard), its large scale use is hampered by high cost, long incubation, problems in decontamination, and intermittent shedding of MAP [28]. Since no single test can identify all the infected animals in a herd at a given time therefore, use of multiple tests have been advocated by various workers [28, 29] for the diagnosis of chronic diseases like JD and comparisons between tests can be done fruitfully (Table 2).

To formulate best strategy for the screening of dairy cattle herds various test combinations were evaluated on different set of samples (Table 3). PCR when used in combination with serum and milk ELISA was able to detect greater number of animals that were infected in comparison to the combined use of microscopy, culture, and ELISA. Low sensitivity of PCR was due to isolation of DNA by manual methods without using kits. Combination of PCR in Ziehl-Neelsen stained smears and PCR in tissues would increase sensitivity of diagnosis [30]. Microscopy and ELISA can also be a good combination to detect MAP in clinical specimen [20, 31]; however, PCR has proved to be highly specific and sensitive in detecting MAP [32, 33]. Among serological tests, ELISA is reported to have better sensitivity than CFT in clinical and subclinical cases, thereby confirming diagnosis 6 to 9 months before excretion of MAP and possible detection by culture [34]. IS*900* PCR has sensitivity and specificity equal or greater than culture and takes only hours as compared to 6–12 weeks by culture [35]. IS*900* sequence for the confirmation of MAP is the most commonly targeted sequence but 100 percent uniqueness of this sequence is doubtful. Potential cross-reactions can result in false-positive results because of the homologous nature of IS*900*-like elements [36]. There was little difference in relative sensitivity of serum ELISA–faecal PCR (83.3%) and milk ELISA–milk PCR (79.3%), though there was significant difference in sample size, 62 and 207, respectively. Therefore, there may be chances of higher agreement of milk ELISA–milk PCR in comparison to serum ELISA–faecal PCR, if evaluated on large sample size. Milk PCR has been reported to be highly sensitive and specific in cattle [37]. High sensitivity of milk ELISA has also been reported [21, 29], though serum ELISA was highly sensitive for detection of MAP in goats, sheep, and cattle [1]. Waters et al. [38] reported that milk ELISA missed 60.0% of true positives which may be due to anergy. No constant association with enhanced antibody responses during development from asymptomatic stage to clinical stage of disease in cattle has been reported [39]. Milk ELISA diagnosed 59.7% of animals that excrete MAP in their feces [40]. Using milk ELISA, Stabel et al. [37] detected 25.0% of cows that were faecal culture positive.

Kappa statistics and calculation of relative agreement between milk ELISA–milk PCR and serum ELISA–faecal PCR showed “substantial agreement” followed by “fair agreement” of serum ELISA–milk ELISA and faecal microscopy–faecal PCR for the detection of MAP infection in different number of samples. A perfect agreement (95%) was also assessed by Duthie et al. [41] between serum and milk ELISA. Lower correlation between serum and milk ELISA may be due to differences in days after parturition [42]. Factors that influence milk quantity and quality may affect correlation between two tests. Very low agreement between two tests has been reported [43] but milk ELISA had higher agreement with culture than serum ELISA. Contrarily, good agreement (Kappa value 0.68) between milk and serum ELISA in dairy goats has been reported [44]. Higher relative sensitivity of serum ELISA with respect to faecal PCR and of milk ELISA with respect to milk PCR showed that serum and milk ELISA followed by milk PCR were the reliable tests for the detection of MAP. Sensitivity of serum ELISA was higher as compared to milk ELISA and PCR; however, potential of milk ELISA as useful and convenient test to estimate herd prevalence was mainly due to suitability of milk as convenient sample and ELISA and PCR can be done on same samples. Milk ELISA was good screening test and milk PCR a good confirmatory test in dairy herds. Repeated testing by an easy and quick test like milk ELISA could be applied in noninfected herds to increase confidence in their status as noninfected [42]. Milk ELISA has been considered as cost effective, accurate, and alternative to faecal culture for the diagnosis of JD in goats [44]. Neither serum ELISA nor faecal culture or PCR have been shown to be effective for the early detection of MAP in dairy cattle [45]. Present study indicated that serial testing is needed for confirmatory diagnosis and milk ELISA followed by milk PCR was sufficiently reliable for the detection of MAP infection in dairy animals. Milk ELISA has been shown to have higher sensitivity than milk PCR [21], whereas other workers reported milk PCR was highly sensitive and specific in dairy cattle [37, 46]. Two new technologies (microfluidics and lab on chip) have been proposed to underpin development of laboratory free diagnostics for the MAP but are in very nascent stage of development [47].

## 5. Conclusion

For the diagnosis of MAP infection, milk microscopy and milk ELISA were good screening tests and IS*900* milk PCR for the confirmation. In different tests used serum ELISA was the most sensitive whereas milk ELISA was the most specific test. Use of multiple tests for the diagnosis was fruitful as compared to single test. Study helped to identify better test combination for the diagnosis of JD in dairy cattle herds in Indian conditions.

## Figures and Tables

**Table 1:** Prevalence of Johne's disease in dairy farms (*n* = 30) using multiple diagnostic tests.

	Microscopic examination	Serum ELISA	Milk ELISA	Milk PCR
Dairy cattle tested	355	315	209	201
Positive	131	49	34	29
Percent prevalence	36.9	15.6	16.3	14.4

**Table 2:** Comparison of different diagnostic test combinations for the diagnosis of Johne's disease.

Sample size	Test combinations		Combinations of tests (%)			
	A	B	A^+^ & B^+^ (TP)	A^−^ & B^+^ (FN)	A^+^ & B^−^ (FP)	A^−^ & B^−^ (TN)
204	Serum ELISA	Milk ELISA	16 (7.8)	18 (18.8)	22 (10.8)	148 (72.6)
68	Faecal Microscopy	Faecal PCR	21 (30.9)	10 (14.7)	16 (23.5)	21 (30.9)
207	Milk PCR	Milk ELISA	23 (11.1)	6 (2.9)	11 (5.3)	167 (80.7)
62	Serum ELISA	Fecal PCR	25 (40.3)	5 (8.1)	5 (8.1)	27 (43.5)

TP: positive in A and B, TN: negative in A and B, FP: positive in A and negative in B, and FN: negative in A and positive in B.

**Table 3:** Comparative diagnostic potential of different test combinations.

Test combinations	Kappa values	Strength of agreement	Relative sensitivity (95% CI)	Relative specificity (95% CI)
Serum ELISA–milk ELISA	0.325	Fair	0.470 (0.297–0.648)	0.870 (0.810–0.917)
Faecal Microscopy–faecal PCR	0.241	Fair	0.677 (0.554–0.788)	0.500 (0.381–0.608)
Milk ELISA–milk PCR	0.682	Substantial	0.793 (0.650–0.890)	0.938 (0.915–0.954)
Serum ELISA–faecal PCR	0.677	Substantial	0.833 (0.719–0.907)	0.844 (0.736–0.913)

<0.20, poor; 0.21–0.40, fair; 0.41–0.60, moderate; 0.61–0.80, substantial (good); and 0.81–100, almost perfect.
